# Non-significant p-values? Strategies to understand and better determine the importance of effects and interactions in logistic regression

**DOI:** 10.1371/journal.pone.0205076

**Published:** 2018-11-26

**Authors:** Zarina I. Vakhitova, Clair L. Alston-Knox

**Affiliations:** 1 Griffth Criminology Institute and School of Criminology and Criminal Justice, Griffith University, Brisbane, QLD, Australia; 2 Arts, Education & Law, Griffith University, Brisbane, QLD, Australia; University of Calgary, CANADA

## Abstract

In the context of generalized linear models (GLMs), interactions are automatically induced on the natural scale of the data. The conventional approach to measuring effects in GLMs based on significance testing (e.g. the Wald test or using deviance to assess model fit) is not always appropriate. The objective of this paper is to demonstrate the limitations of these conventional approaches and to explore alternative strategies for determining the importance of effects. The paper compares four approaches to determining the importance of effects in the GLM using 1) the Wald statistic, 2) change in deviance (model fitting criteria), 3) Bayesian GLM using vaguely informative priors and 4) Bayesian Model Averaging analysis. The main points in this paper are illustrated using an example study, which examines the risk factors for cyber abuse victimization, and are further examined using a simulation study. Analysis of our example dataset shows that, in terms of a logistic GLM, the conventional methods using the Wald test and the change in deviance can produce results that are difficult to interpret; Bayesian analysis of GLM is a suitable alternative, which is enhanced with prior knowledge about the direction of the effects; and Bayesian Model Averaging (BMA) is especially suited for new areas of research, particularly in the absence of theory. We recommend that social scientists consider including BMA in their standard toolbox for analysis of GLMs.

## Introduction

You can know the name of a bird in all the languages of the world, but when you’re finished, you’ll know absolutely nothing whatever about the bird… So let’s look at the bird and see what it’s doing—that’s what counts. I learned very early the difference between knowing the name of something and knowing something. [1]

The use of statistical tests has become an almost automatic response of social scientists when faced with the task of data analysis. As a result, papers now routinely report the results of tests that hold no inferential value for the research hypothesis being studied [2]. There is no doubt that, due to the enhanced features it provides, data analysis using *statistical modelling* will, over time, replace *statistical testing*. This anticipated transition should—as applied disciplines shift to this more informative approach to data analysis—abate the tendency to report irrelevant p-values calculated using a rules-based framework. Instead, models will be used to examine relationships between effects on a deeper level, allowing researchers to create or work within sound theoretical knowledge [3].

While in transition, however, the current debate has focused on the use of p-values in the context of reproducibility of research, rather than a more general discussion on the use of statistical testing. The *Statement on P-values: Context, Process, and Purpose* from the American Statistical Association [4] contends that, while the p-value can be a useful statistical measure, it is frequently misinterpreted when reporting research findings. This frequent misuse of p-values has prompted calls for discouraging their use, and even their complete abandonment [5–9]. However, it is clear that the issue is much wider than statistical testing alone. A number of prominent researchers suggest a more productive response to “the p-value crisis” should be improved statistical education, making the foundations of statistical testing clearer and more accessible to students and researchers [10–12] and promoting the use of statistical modelling.

In social science, the need to consider, discuss and disseminate information on the appropriateness of statistical analyses is a current concern, especially in the era of big data. Many recent publications, such as Psychological Science Under Scrutiny [13], discuss the negative consequences of poor implementation of null hypothesis statistical testing (NHST), p-hacking and an over-emphasis on significance. In the context of social science research, it is important to appreciate that NHST evolved in agricultural research at the time of limited means of computation. When analyzing field trials, the meaning and requirements of these tests effectively represented the experimental outcome [14, 15], whereas observational studies, which are common in social sciences, routinely produce one-off non-repeatable data, which is problematic for a statistical analysis based on NHST [16]. In these research settings, relying solely on conventional NHST and failing to look at the outcomes on a deeper level, will often lead to researchers reporting overestimated (or underestimated) effects and other erroneous conclusions that may hinder the development of sound theories [17].

The aim of this paper is to delve beyond the automated analysis researchers often apply when implementing logistic regression, and to unpack the meaning held in inferences derived from four standard approaches to the statistical modelling of binary data, specifically in regards to the interpretation of interactions. We will first demonstrate that interactions are incorporated on the natural (probability) scale of the data, whether or not they are specified, and that this key feature of generalized linear models needs to be considered in model selection and inference. It would be erroneous to conclude that there is no interaction effect based on the usual Wald test (p-values) given in the summary of the model coefficients. We will further show that the extent of the interaction is difficult to interpret without extensive post-processing of the analytical results. We will then also demonstrate that significance testing based on the Wald statistic (included in all standard logistic regression software) is not always appropriate or enlightening. We will show that the results of the Wald test are unreliable in small samples, and can also display aberrant behaviour when the sample size is large [18, 19].

Similar issues with inferences when using the change in deviance, which is a standard model fit criteria will be illustrated, and we highlight that inferences based on the most parsimonious model can be misleading because they are highly dependant on the structure of the sub-populations contained within the data [20]. By example, we will explain how important interactions can sometimes be represented by a simple main effects model, and that this can lead to erroneous conclusions that the interaction of these effects is not important. Analysis of the logistic regression using a Bayesian framework with vague informative priors is then examined and the advantages of working with posterior distributions of coefficients, linear predictors and fitted values are discussed. Finally, Bayesian Model Averaging (BMA) is considered as an option for researchers who are working in areas of new research where sound theories are yet to be established. We show that BMA in this setting has an advantage in allowing model uncertainty to be considered when assessing the contribution of various effects in the model [21]. In addition, BMA allows researchers to consider a range of models and the probability of interactions being important contributors is incorporated in the modelling outcomes.

## Standard approaches for analyzing binary data

### Generalized linear model for binomial data

The generalized linear model (GLM) is a flexible generalization of ordinary linear regression that allows for response variables that have error distribution models other than a Normal distribution [20]. The GLM generalizes linear regression by allowing the linear model to be related to the response variable via a link function and by allowing the magnitude of the variance of each measurement to be a function of its predicted value.

A generalized linear model for binary data is made up of three components:

1. *Random Component* is the probability distribution of the response variable (Y)
  $$\begin{array}{c} Y_{i}∼\text{Binomial}\left(n_{i},\pi_{i}\right) \end{array}$$
2. *Systematic Component* is formed by the predictor variables (*X*_1_, *X*_2_, … *X*_*k*_) as a combination of linear predictors:
  $$\eta_{i}=\sum_{j=1}^{k}X_{ij}\beta_{j},$$
3. *Link Function* is the link between random and systematic components. In this paper, we will use the *logit* link, commonly used for binary data; however, there are other options available. This link is expressed as
  $$g(\pi_{i})=log\left(\frac{\pi_{i}}{1-\pi_{i}}\right)=\eta_{i}$$

The conventional approach to GLM analysis is to conduct a maximum likelihood estimation of the parameters using a Newton—Raphson or Fisher scoring procedure [20]. This approach assumes that the model parameters (*β*) are constant (fixed), but of unknown value. The data used to construct the model (*x*) are assumed to be a *random* sample from the population. Estimates of the fixed parameters (*β*) are the value that will minimize the residual deviance in the model. That is, we answer the question: given this set of data, what estimate of *β* is most likely. Alternatively, researchers can estimate the model parameters in a Bayesian framework, in which case they will treat the parameters (*β*) as random and estimate a posterior distribution of their plausible values (see Section *Bayesian GLM using vaguely informative priors* for a detailed discussion of this approach).

### Interactions in generalized linear models

**Fig 1:**
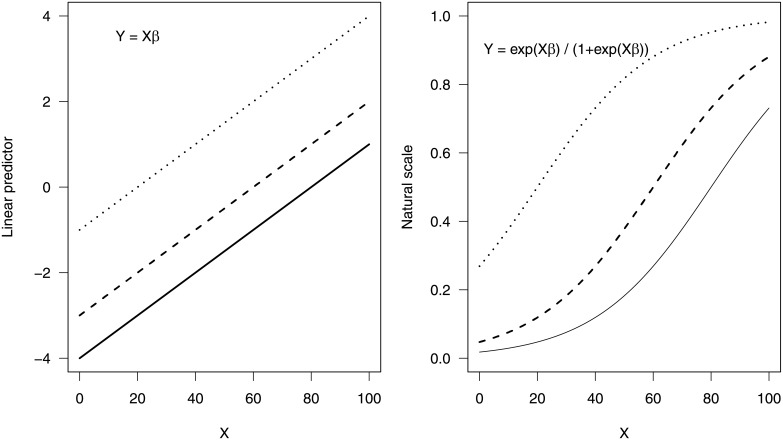
An example of induced iterations in GLM with main effects only. The linear predictor (left panel) shows constant difference between the three lines. On the natural scale (right panel), we see varying distance between the three lines, which is an interaction induced by the back-transformation of the logit model. Here, the solid line is *β* = 1; the dashed line is *β* = 2; and the dotted line is *β* = 3.

The interpretation of interaction effects in generalized linear models is more complex than in basic linear models due to the link function between the systematic component and the response variable [22–27]. The link function in a GLM (except in the case of Normal data) ensures that, when linear predictors are back-transformed to their natural scale, an interaction will be included, whether specified or not [27]. Fig 1 provides a schematic representation of this phenomenon.

Fig 1 (left panel) illustrates a simple linear predictor without the explicitly declared interaction terms in the logistic GLM. We note that the difference between outcomes is constant for all values of *X*. Fig 1 (right panel) shows that on the natural scale the effect of change in *X* on response variable *Y* depends on the value of both *X* and *β*.

When interactions are automatically imposed by the link function, they are not parameterized in the model, so their effect on the overall probability cannot be directly measured or tested using the standard numerical summary for GLM [28–30]. Often, for theoretical reasons, researchers explicitly specify interaction terms. Under these model parameterizations, the presence of both these parameterized interactions and the interactions automatically imposed by the link function complicates the interpretation of the relationship between the response variable and the explanatory variables even further.

To date, the conditional nature of interaction specification is not yet well known and is often overlooked by social scientists [27]. Analysts who fail to recognize the presence of the interactions automatically imposed by the GLM, can inadvertently report misleading inferences. According to Clarke (2006) [31], who surveyed a sample of social science journals, the errors associated with the incorrect interpretation of interaction effects in GLMs are very common.

## Determining the significance of the coefficients in generalized linear models

We will now present four approaches to determining the significance of effects in the GLM using 1) the Wald statistic for hypothesis testing, 2) testing the model terms via model fit using deviance measure, 3) Bayesian GLM using vaguely informative priors and 4) Bayesian Model Averaging analysis. Using the example data, we then demonstrate the limitations of the significance testing based on p-values and show how analyses in the Bayesian framework could help address these limitations.

### Significance testing using the Wald statistic

The Wald test, routinely supplied in statistical summaries of the GLM analysis (using most standard software), tests the null hypothesis that a parameter is equal to zero (*H*_0_: *β*_*j*_ = 0) by comparing the estimated parameters with standard Normal(0, 1). Formally, the Wald test for each parameter of the model is

$$\begin{array}{c} \frac{\left({\hat{\beta }}_{j}-0\right)}{SE\left({\hat{\beta }}_{j}\right)}∼N\left(0,1\right) \end{array}$$ (1)

The p-value of the Wald test then measures the location of the statistic in the N(0, 1) distribution and assigns a probability of seeing a value this extreme or greater under the null hypothesis. Some statistical packages use a different version of the Wald test, relying on $\chi_{1}^{2}$; however, the discrepancies addressed in this paper remain the same.

For logistic GLM, standard software provides the Wald test on the linear predictors, however it has a number of serious limitations. As noted in Agresti (2013) [18], it relies on large samples and performs rather poorly for small samples. Furthermore, the results of the Wald test depend on the scale of the parameterization. That is, on the linear predictor scale, the Wald statistic will yield a conservative estimate, sometimes missing significant effects, and on the natural scale of the data (binary, proportion), the same Wald statistic may be too liberal, signalling significance of effects when that is unwarranted [18]. In addition, the Wald test performs best when the true effect is small or moderate, but can show aberrant behaviour with large effects [19]. Analysts may be unaware that the inferences from their work are subject to any of these issues, as detection of these problems often requires a thorough exploratory data analysis and a range of post-analysis checks.

### Deviance: Significance testing via model fit using deviance

Instead of relying on the Wald test and associated p-values to perform significance tests, an alternative approach is to judge the variable’s true contribution to the model by examining a goodness of fit measure, known as deviance. When assessing models, rather than using deviance as a measure of model fit, it is more useful to compare two nested models. Using this approach, we test whether removing a covariate from the current model significantly worsens the fit [20].

The change in deviance is represented by

$$\begin{array}{c} \Delta D=2(l\left(\pi_{\text{current}};y\right)-l\left(\pi_{\text{reduced}};y\right)) \end{array}$$ (2)

where *l*(***π***_current_; **y**) is the log-likelihood of the currently preferred model (starting the analysis with the saturated model and deleting covariates). Then *l*(***π***_current_; **y**) is the log-likelihood of the proposed reduced model.

To estimate the effects in GLM using this technique, we compare the *change in deviance* (Eq 2) with a $\chi_{1}^{2}$ distribution. If the change in deviance is deemed significant, this is an indication that removing the covariate results in a significantly worse fit of the model to the data. This implies that this effect should be reintroduced into the model.

While GLM analysis using change of deviance is generally used in the literature to determine the model of “best” fit, the analyst needs to consider these results in context. As noted in [20], the change in deviance should be used as a model-building tool, assisting the researcher to find effects that are quite clear, but, when used as a tool for determining the importance of effects, the associated $\chi_{1}^{2}$ comparison (using p-values) should not be taken too literally, but rather as an indicator of potential effects. Essentially, this technique is aimed at finding the most parsimonious model to fit the data, and, as we will show in our following example (see Section *Results using the change in deviance*), much will depend on the sizes of the sub-populations that determine the model parameters and their relative effects.

### Bayesian GLM using vaguely informative priors

An alternative method of estimating the coefficients of a GLM is to take a Bayesian approach to data analysis. In a Bayesian framework, the parameters are considered to be random (as opposed to fixed under the conventional frequentist paradigm), and the resulting analysis provides a probabilistic representation of the parameters known as a *posterior distribution*. The following is a very brief overview of the Bayesian approach to data analysis. For a more detailed introduction to how Bayesian analysis can be utilized in the social sciences we suggest [16, 32, 33].

A Bayesian analysis, using our GLM specification, consists of three basic building blocks:

1. **Prior distribution** of the unknown parameters, *p*(*β*).
2. **Sampling distribution** of the data (also known as the likelihood), *p*(*y* ∣ *X*, *β*). Here *y* represents the response data and *X* represents the covariates.
3. **Posterior distribution** of the unknown parameters, *p*(*β*, ∣ *y*, *X*).

The prior distribution represents the researcher’s belief in regards to the parameter values, *β*_*j*_, before the data are collected [21]. In some cases, the researcher may have no genuine knowledge about the parameters, such as in areas of new research where no theory is yet available. In such a case, analysis is usually conducted using a *non-informative prior distribution* that allows equal probability over all possible values of the parameter. In other cases, such as our example to follow, previous research, or known characteristics of a specific model, allows analysts to specify an informative prior distribution.

A reasonable approach to obtaining a prior distribution for the logistic regression, frequently cited in the literature [11, 34] is to consider the parameters of the GLM (*β*) as having a multivariate Normal distribution, represented in Eq 3:

$$\begin{array}{c} p\left(\beta \right)∼\text{MVN}\left(\mu ,\Sigma^{2}\right) \end{array}$$ (3)

The researcher then needs to nominate either a fixed value for the prior mean(s) (***μ***) and variance (Σ), or some alternative modelling scheme to determine these parameters. In the absence of any prior knowledge, ***μ*** can be set at zero (no effect), and the variance—co-variance (Σ^2^) can be set as a diagonal matrix with large variance.

The posterior distribution of the parameters in the model is estimated using Bayes’ rule:

$$\begin{array}{c} p(\beta |y)\propto p(y|\beta ,X)p(\beta ) \end{array}$$ (4)

The likelihood *p*(*y* ∣ *X*, *β*) is common between frequentist and Bayesian paradigms. In statistical modelling, the choice of likelihood is likely to be the most influential assumption in terms of parameter estimation [33]. It is important to consider the trade-off between the prior distribution and the likelihood distribution of the data. With a large sample size, the likelihood specification will dampen the effect of the prior on the resulting posterior distribution. With small sample sizes, the prior will play a more influential role. In the case of logistic regression, the posterior distribution *p*(*β*, ∣ *y*, *X*) is proportional to the binomial likelihood multiplied by the multivariate Normal prior distribution (Eq 4). A convenient sampling method to estimate these parameters is a Random Walk Metropolis Hastings algorithm. For a detailed description of this estimation method, see [34, 35].

Bayesian inference with vaguely informative priors is a suitable estimation method for logistic GLM, which will yield posterior distributions for modelled parameters. However, in the absence of theory, the researcher will still be required to determine the most appropriate prior distribution. Bayesian Model Averaging is an alternative that requires no special prior knowledge about the direction of effects and interactions, with the added benefit of assisting in model selection.

### Logistic regression using Bayesian Model Averaging

When determining the significance of various effects, the previously discussed Wald tests, changes in deviance and Bayesian estimation only consider uncertainty of the parameter estimates [32]. Bayesian Model Averaging (BMA) is an alternative to the conventional approach of determining the significance of effects associated with individual coefficients where a single model is fitted to the data. BMA considers both the parameter uncertainty and the model uncertainty, and is designed to assist in model selection when theoretical guidance is weak or unavailable. This is often the case in exploratory studies in areas of new research.

The most notable departure of BMA from the conventional approaches to determining the importance of the effects is that we no longer attempt to define one “perfect” model. It assumes that many models are plausible (for example, in a dataset with three binary variables, 2^3^ = 8 models are possible). BMA averages the predictions over all plausible models, taking into consideration the probability of each model being preferred. If a model is very unlikely, it is assigned a lower weight than a well-supported model.

Bayesian Model Averaging has been successfully implemented in many areas of research, including social sciences, economics, ecology, political analysis and criminology [21, 36–40]. The following is a very brief introduction to the BMA procedure.

BMA considers a number of possible models and computes the posterior distribution of coefficients based on weighted averages using the model posterior probabilities. Under the BMA framework, the posterior probability of model *k* (*M*_*k*_) being the favoured model (out of *j* possible models) is represented by Eq 5:

$$\begin{array}{c} p\left(M_{k}|X\right)=\frac{p\left(X|M_{k}\right)p\left(M_{k}\right)}{\sum_{i=1}^{j}p\left(X|M_{i}\right)p\left(M_{i}\right)} \end{array}$$ (5)

where *p*(*M*_*k*_|*X*) is the conditional probability of the model *k* given the observed data *X*, *p*(*X*|*M*_*k*_) is the likelihood of the data under the assumption of model *k* and *p*(*M*_*k*_) is the prior probability of model *k*. When uncertain about the credibility of the model, the prior probability for each model, *p*(*M*_*k*_), is usually taken as a uniform distribution, where all models are considered to be equally plausible. We represent this by Eq 6:

$$\begin{array}{c} p\left(M_{k}\right)=\frac{1}{j} \end{array}$$ (6)

To compare models, the BMA analysis uses a Bayes’ factor (*BF*_*k*_), which, for each model, is the posterior probability of model *k* divided by the sum of these posterior probabilities for all models. As Bayes’ factors can be difficult to estimate [21], the Bayesian Information Criterion (BIC) [41] can be used as an approximate Bayes’ factor in the modelling process. This yields the posterior probability of each model as Eq 7:

$$\begin{array}{c} p\left(M_{k}|X\right)=\frac{exp(-\frac{1}{2}BIC_{k})}{\sum_{i=1}^{j}exp(-\frac{1}{2}BIC_{i})} \end{array}$$ (7)

The BIC for the logistic model is calculated for model *k* as Eq (8):

$$\begin{array}{c} BIC_{k}=D_{k}-df_{k}log\left(n\right) \end{array}$$ (8)

where *D*_*k*_ is the resulting deviance of model *k*, *df*_*k*_ is the degrees of freedom associated with model *k*, and *n* is the sample size in the case of binary data or the total number of subjects if using proportional data. The BIC or the components necessary for its calculation are provided as standard GLM output in most software packages.

Using the BIC, the posterior probability of each model *k* is calculated as per Eq 7. In this BMA approach, the “best” model is the one with the smallest BIC [21]. However, when estimating the parameters, rather than basing decisions on this single best model, researchers can assess the relative merit of each model included in the analysis using the posterior distribution of the parameter. A weighted estimate for the mean and variance of the posterior distribution associated with each model parameter, *β*, can be estimated using Eqs 9 and 10:

$$E(\beta_{k}|D,\beta_{k}\neq 0)\approx \sum_{i\in A_{k}}{\hat{\beta }}_{k}\left(i\right)p\left(M_{i}|D\right)$$ (9)

$$Var(\beta_{k}|D,\beta_{k}\neq 0)\approx \sum_{k\in A_{k}}\left(var_{i}\left(\beta_{k}\right)-{\hat{\beta }}_{k}^{2}\right)p\left(M_{i}|D\right)-E{\left(\beta_{k}|D,\beta_{k}\neq 0\right)}^{2}$$ (10)

The inclusion (or exclusion) of parameters alters the meaning of the parameters contained in each of the models and, as such, the estimated coefficients from BMA are in the form of a posterior distribution. In terms of inference, using BMA, predictions are based on the weighted average of the predictions from the individual models.

Understanding the math behind the BMA procedure, while helpful, is not strictly necessary, as the basic form of the BMA described above is implemented in a number of easy to use software packages. A more detailed discussion of Bayesian model selection for social scientists is given by [21, 42]. [43, 44] provide practical tutorials on implementing the BMA using the “BMA” R package.

We will now demonstrate the methods discussed above using an example study from the area of cyber abuse victimization. The aim of the example is to consider the relative strengths and weaknesses of each analysis in determining the importance of effects and interactions in logistic regression.

## A pedagogical example study: Risk factors for indirect cyber abuse victimization

In this section we illustrate the above discussed methods of determining the importance of effects using a simple example study, which examines the use of indirect methods of cyber abuse. The data used in this example are fully described in [45]. This example study involves GLM where the interaction is explicitly included with main effects. The Wald test (z-scores and associated p-values) produced by the summary output from the saturated GLM indicated no effects from any of the included coefficients, but further investigation revealed issues with these results. We examine several methods for estimating the importance of effects and interactions in GLM. The skeletal R commands [46], necessary to generate these analyses, are also provided.

The main objective of this study was to identify the factors associated with an increased risk of personal victimization from different methods of cyber abuse. This study extended a pilot study [45] that involved a series of 12 qualitative interviews with victims of cyber abuse (see Section Supporting information, S1 File for the list of interview questions). The interviews with victims of cyber abuse reported in [45] were conducted in accordance with the ethical requirements of the Griffith University Human Research Ethics Committee and complied with ethics guidelines set forth by the HREC recommendations. The study was approved by the University’s Ethics Committee (Ethics Approval CCJ/07/14/HREC). Participants were informed that their data would be treated anonymously and that they could terminate the interview at any time without providing any reason. We received oral informed consent (digitally recorded) from all participants before they participated in an interview. The Ethics Committee approved obtaining an oral (digitally recorded) consent rather than written consent in situations where interviews were to be conducted over the phone or Skype. From the outcomes of the pilot study, the researchers hypothesized that the method of abuse used against the victim (direct vs. indirect) was influenced by the offender—victim relationship (no prior relationship vs. prior relationship), the type of offender motivation (expressive vs. instrumental) and/or a combination of both factors.

*Indirect cyber abuse* involves methods of abuse that do not require accessing or contacting the victim directly (e.g. via email or SMS). Examples of indirect cyber abuse include posting derogatory, false or private information about the victim on public websites, social media pages, and other similar methods [45]. *Direct cyber abuse* requires knowledge of the victim’s personal contact information (i.e. e-mail address, mobile phone number, or personal Facebook page); direct cyber abuse is executed through contacting the victim via e-mail, text messages, the victim’s social media pages, surveillance, hacking into the victim’s computer and other such methods.

*Expressive motivation* results from some injury (real or perceived) or ideological disagreement (e.g. disagreement about a divisive or emotionally charged issue, such as race, gender or politics). Cyber abuse events motivated by expressive ends involve offenders acting to express their hostility toward the victim. In contrast, *instrumental motivation* is triggered by more practical needs, such as the desire to change or reinforce the *status quo* in a particular social situation, or in an attempt to establish or re-establish a sexual/romantic relationship with the victim.

The pilot study resulted in the development of the following hypotheses:

*Hypothesis 1:* When there is no prior offender-victim relationship and the motivation is instrumental, indirect abuse is more likely than direct abuse.
*Hypothesis 2:* Indirect abuse is more likely when motivation is instrumental and there is a prior offender-victim relationship compared to when offender-victim relationship is absent and motivation is instrumental.
*Hypothesis 3:* Indirect abuse is more likely when offender-victim relationship is absent and motivation is expressive compared to when offender-victim relationship is absent and motivation is instrumental.
*Hypothesis 4:* Indirect abuse is especially likely, when offender-victim relationship is present and motivation is expressive compared to when offender-victim relationship is absent and motivation is instrumental.

To test the hypotheses, the researchers conducted a quantitative content analysis of a sample of newspaper reports (*n* = 110) that detailed incidents of cyber abuse victimization. The resulting data set contains three binary variables: response variable *Method*, and two predictor variables, *Relationship* and *Motivation*, which were coded to complete the statistical analyses necessary to answer hypotheses 1 to 4 (see Table 1).

**Table 1:** Coding scheme for response and explanatory variables used in GLM analysis of cyber abuse.

Code	Method	Relationship	Motivation
0	Direct	No prior relationship	Instrumental
1	Indirect	Prior relationship	Expressive

Table 2 presents the descriptive statistics of the variables of interest in our Example dataset. The mean observed for each variable indicates that we have a reasonable distribution of cases and, as such, can proceed to the analysis.

**Table 2:** Descriptive statistics for response and explanatory variables in GLM analysis of cyber abuse.

Variables	Range	Mean	SE	*n*
*DV*: Method of cyber abuse	0–1	0.45	0.047	110
*IV*_1_: Offender—victim relationship	0–1	0.57	0.047	110
*IV*_2_: Offender motivation	0–1	0.73	0.042	110

The following Results sections will first describe the exploratory data analyses and then compare the results obtained through a conventional statistical analysis using: 1) the Wald statistic and 2) the change in deviance. We then examine alternative analyses in the form of: 3) the Bayesian GLM, and 4) Bayesian Model Averaging analysis.

## Results

To assist readers in reproducing the results in this section or using the techniques in their own analysis, we provide commented R code for each of the 4 models in Section Supporting information, see S3 File. The complete dataset used in the analyses in the form of csv file is also provided in Section Supporting information, see S2 File.

### Exploratory data analysis

A standard statistical analysis for binary data is generally to use a GLM with a binomial family, and, in this case, we intend to use a logit link function. Before commencing our analysis, in keeping with standard practices [47], we performed some exploratory data analyses (EDA). This step enables us to better understand the results from our analysis and aids in the detection of potential issues.

Table 3 shows the proportion of cases of indirect cyber abuse in total cases in different combinations of our independent variables. Table 3 suggests that cases where both a prior offender—victim relationship is present and motivation is expressive have the highest proportion of indirect abuse (62%), while the scenario where offenders and victims are strangers and the incident is motivated by instrumental ends has the lowest proportion of cases of indirect cyber abuse (22%). This is in line with our expectations based on the analysis of interviews with victims of cyber abuse (see *H*_1_ and *H*_4_). Further, while having a prior offender—victim relationship in the absence of expressive motivation and having an expressive motivation in the absence of a prior offender—victim relationship is associated with a higher proportion of indirect cyber abuse cases, this apparent increase is small (both scenarios are well below 50%), and the sample size for the cases is quite small (*n* = 11 and *n* = 29). As such, we do not expect any statistical models to show strong support for hypotheses *H*_2_ or *H*_3_.

**Table 3:** Proportion of cases of indirect cyber abuse for various combinations of variables.

Coefficients	$\hat{p}$	*N* total	Hypothesis
No relationship, Instrumental motivation	0.22	18	*H*_1_
Relationship, Instrumental motivation	0.27	11	*H*_1_
No relationship, Expressive motivation	0.34	29	*H*_2_
Relationship, Expressive motivation	0.62	52	*H*_3_

#### A single-factor GLM analysis

The purpose of this section is to make the expected outcome clear, so that the following analyses, which are conducted using standard parameterizations of the variables, can be fully explained to the reader. Under normal circumstances, logistic regression models are likely to be much more complex than in our example, so we would not generally factorize multiple variables into a single variable representation as we do here.

As this simple dataset has only two binary predictor variables, an easy method of estimating the combined effect of prior offender—victim relationship and expressive motivation is to convert Relationship and Motivation into a single-factor variable containing four levels, where 1 = Relationship0-Motivation0, 2 = Relationship0-Motivation1, 3 = Relationship1-Motivation0, and 4 = Relationship1-Motivation1. The data can then be analyzed using a generalized linear model with a binomial family and a logit link.

$$\begin{array}{c} \text{logit}\left(Method\right)=\underset{H_{0}}{\underset{︸}{\beta_{0}}}+\underset{H_{1}}{\underset{︸}{\beta_{1}\text{Rel(1)-Mot(0)}}}+\underset{H_{2}}{\underset{︸}{\beta_{2}\text{Rel(0)-Mot(1)}}}+\underset{H_{3}}{\underset{︸}{\beta_{3}\text{Rel(1)-Mot(1)}}} \end{array}$$ (11)

Table 4 presents the usual summary output for the model represented by Eq 11 that is provided by most modern software.

**Table 4:** Summary of generalized linear model of indirect cyber abuse using a single factor with four levels (Eq 11).

Coefficient	Estimate	Std. Error	z-value	Pr(> |*z*|)	Hypothesis
Intercept (*β*_0_)	-1.25	0.57	-2.21	0.03 **	*H*_1_
Relationship (*β*_1_)	0.27	0.88	0.31	0.76	*H*_2_
Motivation (*β*_2_)	0.61	0.69	0.89	0.37	*H*_3_
Relationship+Motivation (*β*_3_)	1.72	0.63	2.72	0.01 ***	*H*_4_

Note:

**p<0.05;

***p<0.01

Table 4 provides the summary output when the generalized linear model is fitted as per the factorization given in Eq 11. To gain a complete understanding of the inference we can obtain from the analysis, it is important to consider both the *absolute* and the *relative effects* of the model. The absolute effect is the change in the probability of the outcome, dependent on all of the parameters. The relative effect is the proportional change induced by a change in predictor. In this model, *β*_0_ is known as the intercept. This is the absolute effect of instrumental motivation and no prior offender—victim relationship (on the linear scale). We note that the Wald test quite convincingly discounts the null hypothesis that *β*_0_ = 0, with a p-value of 0.03. In practical terms, as this coefficient is less than zero, this implies that the probability of the offence using indirect methods of cyber-abuse under these circumstances is likely to be less than 0.5 (which supports hypothesis *H*_1_). To understand this effect, we need to consider the relative effect—in this case, the point estimate of the probability of the use of indirect methods. This relative effect is calculated using transformation to the natural scale of the data $\frac{\exp(-1.25)}{1+\exp(-1.25)}=0.22$, and is around the value expected from Table 3.

The coefficient related to our second hypothesis (*H*_2_), which theorized that a prior relationship between the offender and victim (but still an instrumental motivation) will lead to an *increased* probability of the cyber abuse being indirect in nature, is given as *β*_1_ (Table 4). The value ${\hat{\beta }}_{1}=0.27$ can be viewed as the increase in odds of abuse by indirect method happening by the addition of a prior relationship. This is assessed using the exponent value of the coefficient, exp(0.27) = 1.31. This value implies that the odds of abuse via an indirect method increase by 31% with this changed victim—offender relationship (using the absolute effect), and as such supports hypothesis 1 (*H*_2_). However, the relative effect of an increase of 31% when indirect methods were a relatively rare 22% only leads to a probability of indirect method under this victim—offender scenario of 27% (also as expected from preliminary data analysis, Table 3). While this seems to eliminate hypothesis *H*_2_, this is only a point estimate. The accompanying z-score is used to test the null hypothesis that *β*_1_ = 0, and returns a p-value of 0.76. This implies that we cannot reject the null hypothesis. However, as we will expand on this point in Section *Results using the Wald test*, rejection of a null hypothesis is not sufficient reason to discount the possibility of a true effect, and more efficient modelling techniques are available to quantify the effect of a variable.

The third hypothesis (*H*_3_) (expressive motivation increases the probability of indirect cyber abuse) is tested via estimates of ${\hat{\beta }}_{2}=0.61$. Once again, the odds of an increase in the use of indirect methods via a change in motivation are exp(0.61) = 1.84, which equates to the indirect methods of abuse being 84% more likely in absolute terms. In relative terms, this yields a point estimate for the probability of indirect abuse of 34.5%, however, once again, the Wald test fails to reject the null hypothesis that this additional effect may be zero.

The fourth hypothesis (*H*_4_) has very strong support. From the results in Table 3, we note that the increase in odds of indirect methods of cyber abuse when the victim and offender are known to each other and the offenders motivation changes to expressive is exp(1.72) = 5.58. The relative effect of this is that the probability of the use of indirect methods under this scenario is 62%. In addition, the p-value used to test the null hypothesis of no effect (*β*_3_ = 0) is soundly rejected, with *p* = 0.01. This is in line with expectations based on the results from Table 3.

In this analysis, the matching of the levels of the single factor to the four research hypothesis has ensured that the p-values reported in Table 4 are of inferential value. If a different contrast scheme had inadvertently been applied, these p-values would not be useful in discussing the outcome of the analysis in terms of the hypothesis.

From this simple single factor GLM analysis, we now expect that a more conventional data analysis, in which Relationship and Motivation are treated as separate variables, will yield inferences that indicate: 1) having either Prior Relationship or Expressive Motivation (but not both combined) may result in an increased use of indirect methods of cyber abuse, but the evidence is not strong; 2) the combination of both Prior Relationship and Expressive Motivation is very likely to result in a higher probability of using indirect methods of cyber abuse, and the outcome of statistical analysis should strongly support this.

### Results using the Wald test

To determine the importance of effects and interactions in this example analysis, we now model the data following a more conventional approach:

$$\begin{array}{c} \text{logit}\left(Method\right)=\underset{H_{1}}{\underset{︸}{\beta_{0}}}+\underset{H_{4}}{\underset{︸}{\underset{H_{2}}{\underset{︸}{\beta_{1}\text{Relationship}}}+\underset{H_{3}}{\underset{︸}{\beta_{2}\text{Motivation}}}+\beta_{3}\text{Relationship:Motivation}}} \end{array}$$ (12)

Using the contrast scheme supplied in Table 1, the intercept, *β*_0_, is the linear predictor (absolute effect), which represents the effect of *No prior offender—victim relationship (Relationship = 0)* and *Instrumental Motivation (Motivation = 0)* on the method of abuse (response variable). Using this model, *H*_2_ is tested by the departure of *β*_1_ from zero and *H*_3_ is tested by the departure of *β*_2_ from zero. However, *H*_4_ is determined using the addition of terms as indicated in Eq 12.

The following R code was used to generate the GLM analysis used in this section:

exampleGLM = glm(formula = Method ~ Relationship * Motivation,

family = “binomial”, data = …)

summary(exampleGLM)

Most standard statistical software, including R, routinely produces the p-values based on z-scores (Wald statistic) when analysis is performed using the glm() function. Table 5 presents the summary output from the glm() function for the full model (Eq 12). If we compare the estimates from Table 5 with those of the single factor GLM (Table 4), we can see that the point estimates, standard errors and the Wald test results are unchanged for the intercept and the main effects (which relate to having only Prior Relationship or only Expressive Motivation). The p-values reported in Table 5 associated with the main effects have inferential value in assessing the first three research hypotheses. However, as *H*_4_ is now assessed using the sum of coefficients $\sum_{i=1}^{3}\beta_{i}$, Table 5 does not provide the associated Wald test, and this p-value is of no inferential value for the fourth hypothesis. We note that $\sum_{i=1}^{3}\beta =1.72$, as per our previous model (Table 4); therefore, while the results are yielding an indication of the interaction effect, the standard summary Wald tests are not able to measure this term.

**Table 5:** Summary of coefficient estimates for full GLM model (Eq 12).

Coefficient	Estimate	Std. Error	z-value	Pr(> |*z*|)	Hypothesis
Intercept (*β*_0_)	-1.25	0.57	-2.21	0.03 **	*H*_1_
Relationship (*β*_1_)	0.27	0.88	0.31	0.76	*H*_2_
Motivation (*β*_2_)	0.61	0.69	0.89	0.37	*H*_3_
Relationship:Motivation (*β*_3_)	0.84	1.01	0.83	0.40	≠ *H*_4_

Note:

**p<0.05

In this example, the p-value using the Wald test to measure the effect of Prior Relationship and Expressive Motivation individually (but not in combination) appears to be non-significant, suggesting that we cannot reject the null hypothesis of no added effect for these terms. Additionally, it is worth further emphasizing that, if the coding of Relationship and Motivation in Table 1 was switched, both main effects would be deemed significant (data not included) however, these p-values would be testing a different “hypothesis” in which the presence of an offender—victim relationship and expressive motivation is the base level of comparison.

The next step that analysts often take is to drop the higher order terms, which, in this case, is the interaction term, and re-run the model. To avoid duplication, results of this analysis will be incorporated into the change of deviance approach (Section *Results using the change in deviance*).

In summary, the Wald test, while convenient (as it is routinely supplied in most standard statistical software (e.g. SPSS, Stata, R)), and reliable with large samples, when used in GLMs with interactions can produce results that are difficult to interpret.

### Results using the change in deviance

As an alternative to determining the importance of effects using the Wald statistic, analysts often use another conventional method, which considers the model fit, known as *the change in deviance criteria*.

Table 6 presents the change in deviance as we sequentially remove terms, starting with the full model. Terms are removed as follows: 1) the interaction term, 2) the motivation term, and 3) the relationship term (motivation is reintroduced).

**Table 6:** Summary of model testing using the change in deviance. Significant outcomes indicate that the dropping of a term results in a significantly worse fit.

#	Model	Deviance	df	Δ Deviance
Model 1	Full model (Eq 12)	138.62	106	
Model 2	Interaction removed (main effects only)	139.32	107	-0.71
Model 4	Motivation removed	144.23	108	-4.92**
Model 5	Relationship removed	143.81	108	-4.49**

Note:

**p<0.05

Table 6 suggests that dropping the interaction from the model does not significantly affect the model fit (p > .10). However, dropping either main effect (motivation or relationship) results in significantly worse model fit (*p* < 0.05). Based on the analysis of deviance, the final model would include both main effects, but not the interaction term.

Based on the results of the single-factor analysis (see Section *A single-factor GLM analysis*), these outcomes are perplexing. The magnitude of the effect for the situation, where both Prior Relationship and Expressive Motivation were present (Table 4) was by far the largest, whereas, in the same model, the effects of only Prior Relationship or Expressive Motivation, while positive, were unconvincing. As such, we were anticipating coefficients that were reasonably small (albeit positive) for both main effects, and a positive coefficient of larger magnitude to be added when both Prior Relationship and Expressive Motivation were present (as per Eq 12). As we can note in Table 7, the addition of the main effects yields a coefficient *β*_0_+*β*_1_+*β*_2_ = 0.41, which is very close in absolute terms to the full model representation (Table 5) $\sum_{i=0}^{3}\beta_{j}=0.47$, which is exactly the same point estimate as the simple single-factor GLM (see Table 4). In terms of relative effect, the expected proportion of indirect method in the full model was 62%, and in the main effects-only model, this estimate was 60%. The p-values reported in Table 7 no longer address the research hypothesis regarding the main effects, as the coefficients relating to relationship and motivation no longer represent *H*_2_ and *H*_3_. These coefficients are now based on data that have both a prior offender—victim relationship and expressive motivation. Using these p-values may lead researchers to erroneous conclusions in terms of *H*_2_ and *H*_3_.

**Table 7:** Summary of coefficient estimates for the main effects GLM. This model was determined using change in deviance (see Table 6).

Coefficient	Estimate	Std. Error	z-value	Pr(> |*z*|)	Hypothesis
Intercept (*β*_0_)	-1.54	0.49	-3.17	0.00***	*H*_1_
Relationship (*β*_1_)	0.92	0.42	2.19	0.03**	≠ *H*_2_
Motivation (*β*_2_)	1.03	0.50	2.05	0.04**	≠ *H*_3_

Note:

**p<0.05;

***p<0.01

Both the Wald statistic and the change in deviance test are seemingly favouring the main effects model, whereas from our EDA and single-factor GLM, we were expecting an interaction term to be supported (see Sections 1 and *A single-factor GLM analysis*). The relatively small sample size and the chosen parameterization will affect the model selection based on the changed deviance or other test statistics that are searching for the single best model, as they will tend to favour the most parsimonious solution [18]. At a minimum, this problem in small (and moderate) samples suggests that p-values associated with the change in model deviance should be used as a guide only, and that researchers should look more closely at other summary statistics, such as effect size and confidence intervals.

**Fig 2:**
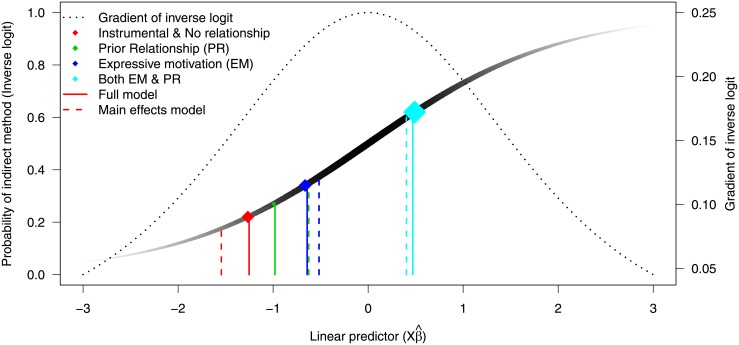
Change in deviance test applied to pedagogical example. The gradated grey line represents the back- transformation of the logit function (inverse logit). The grey levels in this line indicate the gradient of the function, with darker areas having the steepest slopes. The gradient is also represented by the black dashed line and the right axis. The coloured points indicate various subgroups and the size of these points represents their sample size. Solid coloured lines represent the linear predictors obtained from the full model including an interaction term. The coloured dashed lines represent linear predictors from the main effects model.

Looking more closely at this particular example, it becomes clearer why the change in deviance test is favouring the main effects model over the model that includes an interaction. Fig 2 provides a visual representation of the behaviour of linear predictors from the logistic model in this example. The value of the linear predictors is given on the x-axis, the solid graduating coloured line indicates the inverse logit transformation (left y-axis). The thickness and colour graduation of this line reflects the gradient of the inverse logit, with the more prominent the line, the greater its gradient, and hence changes in deviance will be more influential. The dashed line is the gradient of the inverse logit (right y-axis) over the values of the linear predictors, and we observe the gradient is largest at $X\hat{\beta }=0$. From Fig 2, we note that, in general, the linear predictors that would be most affected by a change in fitted value are those for which the probability of an event is 0.5, and that, as the linear predictor approaches an absolute value of 3, changes in its value have a very small effect on the predicted probability of an event (left y-axis).

In this example, there is a relatively large sub-population of the data that has both a prior relationship between the victim and offender and an expressive motivation behind the abuse (*n* = 52). This sub-population is also in an active zone of the inverse logit, with a probability of indirect method of 0.62 (cyan lines in Fig 2). Because of the location and size of this subgroup, a change in linear predictor will have a considerable effect on model fit, and the ensuing change of deviance test. However, as we can see in both Fig 2 and Table 8, the addition of the main effects results in a linear predictor that is only slightly lower than the same term in the full model (change of 0.06). The interaction is naturally represented in the inverse logit and, as such, this main effects model is still capable of representing this effect adequately.

**Table 8:** Results of determining model using change in deviance.

Profile*	Linear Predictor (LP)†	Probability of IM‡	Gradient Inverse Logit	n	LP§	Change in LP
1	-1.25	0.22	0.17	18	-1.54	0.29
2	-0.98	0.27	0.19	11	-0.63	-0.35
3	-0.64	0.34	0.22	29	-0.51	-0.12
4	0.47	0.62	0.24	52	0.40	0.06

Note:

*Offender-victim profile: 1 = Instrumental / No Relationship, 2 = Instrumental / Relationship, 3 = Expressive / No Relationship, 4 = Expressive / Relationship

^†^Full model

^‡^IM–indirect methods of cyber abuse

^§^LP–linear predictor–main effects only

We can also observe (Red and green lines in Fig 2 & Table 8) that the largest impact, in terms of the linear predictor, by dropping the interaction term, is seen in the predictions for relationship only (green lines) and for the subgroup associated with instrumental motivation and no prior relationship (red lines). In terms of the main effect for relationship, we see that it is a small subgroup (*n* = 11) and the gradient on the inverse logit is 0.19. This enables this linear predictor to “move” a reasonable amount and not affect the overall fit to the extent that the change in deviance test will reject the simpler model.

It is now clear that the main effects model will represent the interaction well, and the major concern is that using the Wald test and the change in deviance test could result in the analyst believing they have sufficient grounds to report a statistically significant effect of both relationship and motivation on an individual basis, and overlook the naturally incorporated interaction effect in the GLM. Whereas, thoughtful reflection on the intended meaning of this model would yield a cause for caution in making an inference with regards to either relationship or motivation alone.

In more complex models, particularly high-dimensional data with multiple sources of interactions, it would become increasingly difficult for the analyst to examine the outcomes of this approach. The change in deviance using the model fit is commonly used, however it should be treated with caution, especially in models with explicitly declared interactions. As encapsulated in Fig 2, this method is likely to favour the most parsimonious model and therefore will tend to smooth over effects that relate to subgroups with small membership, along with groups that have high and low probabilities of an event (i.e. events with larger absolute values in terms of the linear predictor).

### Results using Bayesian GLM with vaguely informative priors

**Fig 3:**
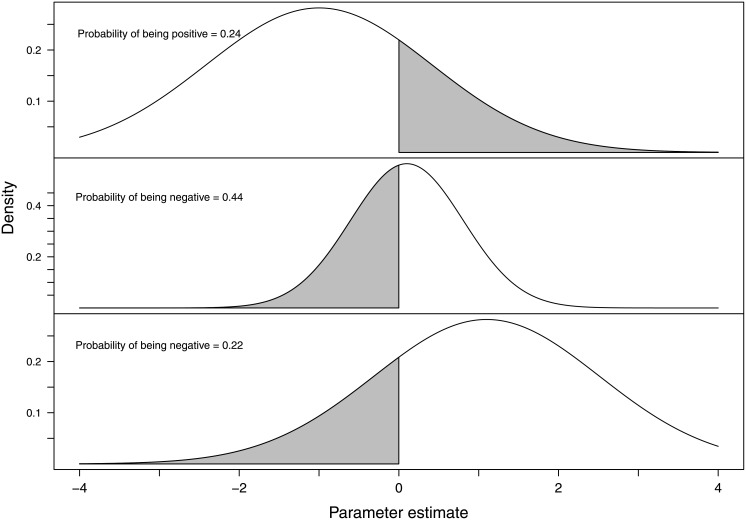
Three vague Normal priors used to compute Bayesian logistic regression. Top: Prior distribution used for the intercept parameter *β*_0_ (Eq 12), which represents persons with no prior relationship and instrumental motivation (*N*(−1, 2)). Middle: Prior distribution used for parameters *β*_1,2_, which represents the additional effect of persons with either a prior relationship OR an expressive motivation over the intercept value (*N*(0.1, 0.5)). Bottom: Prior distribution chosen to represent *β*_3_, the additional effect of both having a prior relationship and expressive motivation (*N*(1.1, 2)). Grey areas represent regions allowed in the prior that are the opposite of our hypothesis.

A Bayesian approach to logistic regression using vaguely informative priors requires the researcher to provide prior distributions of the variables of interest. Novice researchers are often daunted by the concept of providing a prior distribution, and, as a result, often choose very vague priors centred on zero. However, in simple (low-dimensional) models such as our Example study, it is surprisingly intuitive to formulate vaguely informative priors that reflect the researcher’s current state of knowledge. Fig 3 illustrates the prior distributions we use in this analysis.

The prior distributions in Fig 3 were determined using the results of the pilot study (as evidenced by the hypotheses), and we decided on a set of prior distributions as follows:

1. The parameter *β*_0_ (intercept, Eq 12) will most likely be a negative value, as there is no obvious reason for the perpetrator who does not know their victim and is motivated by instrumental ends to resort to indirect methods of abuse. However, as this is research in an area without firm theory, it is prudent to choose a prior that reflects some uncertainty, so a *N*(−1, 2) is used, which has 24% of its density in the positive region (Fig 3, top).
2. The main effects of the existence of a prior relationship and instrumental motivations (*β*_1_, *β*_2_) were thought most likely to be positive, and this is reflected in *H*_2_ and *H*_3_. On reflection, it was not known how large these effects were likely to be, so *N*(0.1, 0.5) priors distributions were considered to adequately reflect the anticipated effect of these variables, with 44% of the distribution contained in the negative region, and potential values of the coefficient contained predominantly in the range ±2 (see Fig 3, middle).
3. Based on the pilot study, *H*_4_ was thought to have the biggest potential effect on the probability of the cyber abuse being conducted via indirect methods, where there was a prior relationship between offender and victim and the offenders motivation was expressive. A *N*(1.1, 2) was chosen to reflect this belief, where the density is predominately in the positive region, with a still large probability of a value from the distribution being negative (22%), as illustrated in Fig 3 (bottom). The prior distribution of the parameters under this formulation is then:
  $$\begin{array}{ccc} p(\beta ) & ∼ & MVN(\mu ,\Sigma^{2}) \end{array}$$
  $$\begin{array}{ccc} \mu & = & \left[-1.0,0.1,0.1,1.1\right] \end{array}$$
  $$\begin{array}{ccc} \Sigma^{2} & = & \left[\begin{array}{cccc} 2 & 0 & 0 & 0\\ 0 & .5 & 0 & 0\\ 0 & 0 & .5 & 0\\ 0 & 0 & 0 & 2 \end{array}\right] \end{array}$$

To estimate the effects of individual coefficients in a Bayesian framework, we compute posterior probabilities associated with the hypothesis of interest, which are akin to p-values in the conventional approach. Several R libraries contain functions to estimate Bayesian logistic models. In this analysis, we used the routine MCMClogit() from the library MCMCpack [48]. Example code for the logit model is shown below:

library(MCMCpack)

ExampleMCMC.glm = MCMClogit(Method ~ Relationship * Motivation,

          burnin = 10000, mcmc = 110000,

          thin = 1, tune = 1.1, b0 = c(-1,0.1,0.1,1.1),

          B0 = c(0.5,2,2,0.5), data = …)

summary(ExampleMCMC.glm)

Here we specify the hyper-parameters of the prior (Eq 3), where b0 represents the Normal means (*μ*_*j*_) and B0 is known as the precision parameter. Precision is the inverse of the variance (Σ^2^) and is sometimes used as an alternative parameterization. Additionally, it is important for readers to be aware of the tune argument. By default, it is set at 1.1; however, users may need to alter this to influence the acceptance rate of the sampler, which should generally be between 0.2 and 0.5 [49, 50]. By using the default tuning parameter of 1.1, we achieved an acceptance rate of 0.34.

Table 9 presents a summary of results from the Bayesian logistic model. To sample the posterior distributions, we executed 100,000 iterations after a burnin period of 10,000, the chains were thinned and every 100^th^ iterant was kept to calculate posterior estimates. In a Bayesian analysis, the whole of the posterior is considered to be the “estimate” of the parameter. As such, plausible ranges of potential values are often considered using various quantiles, which can be used to form credible intervals. In Table 9, a 95% CrI would contain the posterior draws above the 2.5% quantile and below the 97.5% quantile, effectively excluding the 2.5% highest and lowest samples.

**Table 9:** Summary statistics of Bayesian GLM using vaguely informative priors.

Coefficient	Mean	SD	Quantiles				
			2.5%	25%	50%	75%	97.5%
(Intercept)	-1.10	0.40	-1.88	-1.37	-1.09	-0.83	-0.35
Relationship	0.09	0.51	-0.96	-0.25	0.11	0.43	1.05
Motivation	0.34	0.45	-0.51	0.05	0.33	0.65	1.22
Relationship:Motivation	1.16	0.61	0.09	0.73	1.13	1.57	2.35

From Table 9, we note that the coefficient for the interaction term Relationship:Motivation is large in absolute terms (*β* = 1.16), and zero is not included in its plausible range. This result clearly supports research hypothesis *H*_4_. Both main effects, Motivation and Relationship, have a point estimate that is small in magnitude, with the Relationship coefficient having a point estimate close to zero along with a wide plausible range. Although the absence of zero in a credible interval clearly indicates that a coefficient is unlikely to result from a variable with no effect on the outcome, caution should be exercised when considering terms that contain zero in the credible interval. The posterior distribution of the coefficients is a multivariate Normal, and therefore the coefficients cannot be assessed independently. As such, we cannot discount research hypothesis *H*_2_ and *H*_3_ based solely on the inclusion of zero in their credible intervals.

The standard errors from the Bayesian analysis are smaller than the ones generated by the standard glm() function (see Table 5). Overall, the indications of this analysis are in line with the single variable glm (Table 4): indirect methods are unlikely when motivation is instrumental and no prior relationship exists, and indirect methods are most likely to occur when the motivation is expressive and a prior relationship is present. The results of prior relationship or expressive motivation only are indicative of the more likely use of indirect methods but are not, using this model and data, well supported.

**Fig 4:**
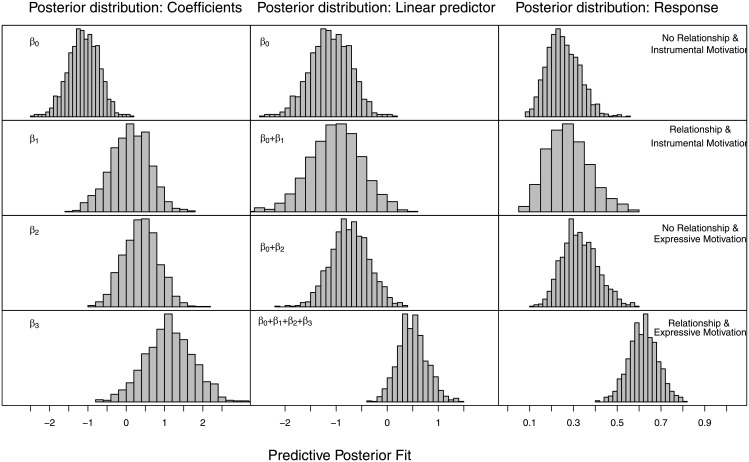
Posterior distributions of coefficients (left column), linear predictors (middle column) and predictive fits (right column).

The outcomes presented in Table 9 are perhaps more easily understood by viewing the posterior samples in Fig 4, which presents posterior distributions of the coefficients in the left hand column. Viewing these, we note that the simulated posterior draws for the interaction coefficient has very few negative values, and, while generally positive, the two main effect coefficient posterior simulations have a reasonable mass of negative samples.

One of the advantages of Bayesian analysis is that it allows us to use posterior distributions of parameters to form posterior distributions of any function of those parameters. Using this property, the linear predictors (Fig 4, middle column) and the predicted responses (Fig 4, right column) can be represented using a predictive posterior distribution, and, from this, credible intervals and plausibility can be considered. This allows the researcher to explore more complex questions, such as extensions in our stated hypotheses, by computing the actual probabilities of interest. Additionally, posterior distributions for the linear and response predictors are not affected by coding or contrast choice.

From Fig 4 and Table 9, we note that the combination of prior offender—victim relationship and expressive motivation is clearly more likely to be associated with the use of indirect methods of cyber abuse victimization (0.62; 80% CrI (0.53, 0.70)), while the outcome that is attributable to prior relationship (0.27; 80% CrI (0.16, 0.40)) but instrumental motivation is very similar to the base level of no prior relationship and instrumental motivation (0.26; 80% CrI (0.17, 0.35)). These results are not supportive of research hypothesis *H*_2_ or *H*_3_. Here, 80% CrI denotes the Bayesian credible interval, in which the highest and lowest 10% of draws from the posterior distribution are excluded [11].

In summary, Bayesian GLM with vaguely informative priors does not rely on large sample asymptotics and is well suited for low-dimension models and when the researcher has at least some idea of the direction of the effects (positive or negative). However, Bayesian GLM requires some prior knowledge and some expertise in its implementation. When analyzing exploratory studies, where prior knowledge may be limited or lacking completely, non-informative priors are generally used, at least in an initial analysis. The Bayesian approach yields the advantage of being able to better respond to the research hypothesis by using the posterior distributions to make direct statements about quantities of interest.

### Results using Bayesian Model Averaging

To demonstrate how Bayesian Model Averaging analysis could be used in the context of GLMs, we utilize the BMA library [51] in R. The following R code was used to generate the summary output from the BMA analysis:

library(BMA)

exampleBMA = bic.glm(form = Method ~ Relationship * Motivation,

       glm.family = binomial(), data = …)

summary(exampleBMA) #tabular output

imageplot.bma(exampleBMA, …) #graphic output

The summary of results from the BMA analysis is presented in Table 10, which contains posterior means, standard deviations and inclusion probabilities for the coefficients associated with each variable. As Table 10 suggests, the inclusion probability for the coefficient containing prior offender—victim relationship (Relationship = 1) and expressive motivation (Motivation = 1) provides strong evidence for indirect methods of abuse, with a posterior mean of 1.07 and estimated probability of 0.8 that this coefficient is not zero. Evidence in favour of *H*_2_ and *H*_3_—that, individually, prior relationship or expressive motivation are more likely to result in indirect methods of abuse—is less compelling. The inclusion probability that either of the coefficients associated with this effect is not zero is 0.20 and 0.22, respectively, and the magnitude of the posterior mean is relatively small. Model 1 (Table 10), which contains only the intercept and a variable consisting of the combination of Prior Relationship and Expressive Motivation (coefficients containing Prior Relationship only and Expressive Motivation are not included in the model), is clearly the most favoured model with a model posterior probability of 0.65.

**Table 10:** Summary statistics from the Bayesian Model Averaging analysis*.

Coefficient	Pr(*β* ≠ 0)^†^	*β*	SD	Model 1	Model 2	Model 3	Model 4	Model 5
Intercept (*β*_0_)	1.00	-0.96	0.38	-0.88	-1.15	-0.86	-1.54	-0.86
Relationship (R) (*β*_1_)	0.20	0.13	0.43	-∓	-	1.08	0.92	-0.12
Motivation (M) (*β*_2_)	0.22	0.19	0.46	-	0.50	-	1.03	-
R-M (*β*_3_)	0.80	1.07	0.67	1.35	1.11	-	-	1.45
Posterior Probability				0.65	0.09	0.08	0.07	0.06
BIC				-368.19	-364.24	-363.84	-363.63	-363.52

Note:

*Best five out of six selected models (cumulative posterior probability = 94%)

^†^ Indicates the inclusion probability of each coefficient

^∓^ Indicates the coefficient is excluded from model

**Fig 5:**
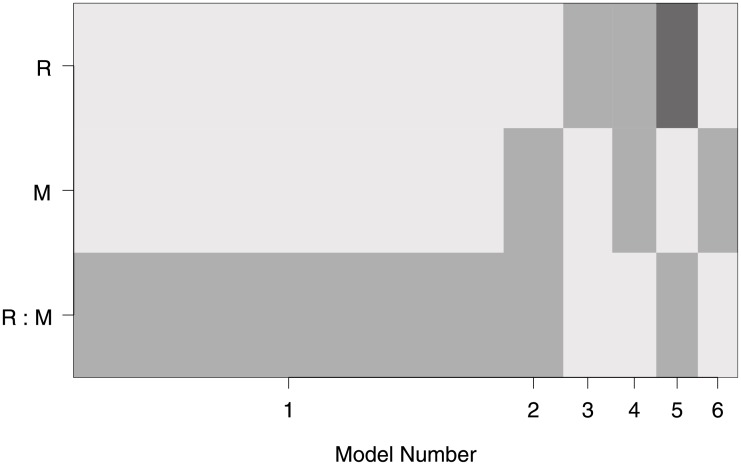
A graph of the selected models explaining the use of indirect methods of abuse using Bayesian Model Averaging analysis. Width of individual rectangles reflects the posterior probability of each model being correct.

Fig 5 presents a graphical representation of BMA information given in Table 10. The width of the individual rectangles is determined by the posterior probability of each model (Table 10). The intercept is always included in the model and as such is not displayed. The medium shade of grey indicates that the coefficient is positive, and dark grey indicates a negative coefficient (light grey indicates the coefficient is not included in the model). As Fig 5 suggests, a model consisting of only the additional effect of a combination of prior offender—victim relationship and expressive motivation is by far the most probable (as indicated by the widest rectangle). The next five best models are all quite similar in terms of their model posterior probability ranging between 6% and 9%, and so, appropriately, this visual representation does not aid in distinguishing these. Interestingly, the model that contains main effects only (Model 4 in Fig 5) is quite unlikely with a posterior probability of only 7%, and the model that contains both main effects and the interaction is not even one of the five best models with posterior probability of less than 3%. Based on these results, it appears that neither the saturated model we chose for interpreting the effect of offender—victim relationship and offender motivation (see Table 5) nor the model chosen using deviance criteria or the Wald test (Table 7) is likely to adequately represent the information contained in these data. However, the Bayesian analysis (Table 9) has captured the salient information within a single model.

### Comparison of inferences from different methods

Table 11 illustrates that the posterior distribution of the BMA coefficients is quite similar to that obtained using Bayesian GLM with vague prior distributions. The coefficients in the standard GLM would yield a similar fitted value for the combined effect of Prior Relationship and Expressive Motivation when the coefficients are summed; however, the magnitude of the coefficients associated with the main effects is more influential. The standard errors in the ordinary GLM tend to be larger, indicating a greater variability in the estimates. The reduction in the magnitude of standard errors between ordinary GLM and the Bayesian estimates suggests that Bayesian GLM could be a good alternative to the standard GLM in the logistic regression model with small sample size when researchers are able to use a vaguely informative prior. Furthermore, both Bayesian GLM with vague priors and BMA produced results that are very similar to each other in terms of both the magnitude and the direction of individual coefficients of interest and the associated standard errors.

**Table 11:** Comparison of coefficients and standard errors from standard GLM, Bayesian GLM posterior estimates (using vague priors) and GLM posterior estimates via BMA.

Coefficient	${\hat{\beta }}_{GLM}$(SD)	${\hat{\beta }}_{Bayesian}$(SD)	${\hat{\beta }}_{BMA}$(SD)
Intercept	-1.25 (0.57)	-1.10 (0.40)	-0.96 (0.38)
Relationship	0.27 (0.88)	0.09 (0.51)	0.13 (0.43)
Motivation	0.61 (0.69)	0.34 (0.45)	0.19 (0.46)
Relationship-Motivation	0.84 (1.01)	1.16 (0.61)	1.07 (0.67)

Based on the results of the BMA analysis, which are consistent with the findings of the single-factor GLM (see Section *A single-factor GLM analysis*) and the Bayesian GLM with vaguely informative priors (see Section *Bayesian GLM using vaguely informative priors*), we conclude that having a combination of prior offender—victim relationship and expressive offender motivation is the scenario in which the use of indirect methods of cyber abuse is most likely to occur. These findings are, on face value, in contrast to the results of the Wald test and the change in deviance method. However, on closer examination of the results of the Wald test and the change in deviance approach, it becomes evident that, when we fully appreciate the implicit interaction within the GLM, along with an understanding of the statistical algorithms and the data, all of the results have similar inferences.

As our example dataset includes a small number of binary variables and, therefore, a relatively low number of possible models, we used the BMA library [51] in R [46], where parameters are estimated using the standard approach using maximum likelihood, and the BIC for each model is calculated using Eq 8. Model prior probabilities are taken as being Uniform (Eq 6).

When the number of variables in the model increases (it is not uncommon for social science research to have 25, 30 or even more variables), the comparison of each possible model can be computationally unfeasible. In such a situation, there are several approaches in the literature to solve this issue. [51] implement a leaps and bounds algorithm [52] to determine a subset of plausible models without fully estimating every possible model.

In summary, Bayesian Model Averaging analysis accounts not only for parameter uncertainty, but also for model uncertainty; it does not rely on large sample asymptotics; and it can be readily used with models with a large number of variables and higher-dimension models.

## Simulation study

As mentioned earlier in this paper, one of the issues potentially affecting the results of this example study is the small sample size, particularly in regards to the sub-populations represented by prior offender—victim relationship and offender motivation. To consider the sample size effects on the results of ordinary GLM and to gain an understanding of the likely behaviour of BMA, we conducted a small simulation study. In the study, we used three sample sizes (*N* = 110, 500, 1000), and sampled 1000 simulations for each. In this simulation study, we have attempted to mirror our example data by setting the true proportions of each scenario of offender—victim relationship and offender motivation, along with method of abuse, as given in Table 12.

**Table 12:** Proportions used for simulations of population in each Rel-Mot†subgroup and method of abuse.

Rel-Mot Subgroup	Proportion in Rel-Mot Subgroup	Proportion of Indirect Method
1: Rel(0)Mot(0)	0.16	0.25
2: Rel(1)Mot(0)	0.10	0.25
3: Rel(0)Mot(1)	0.26	0.40
4: Rel(1)Mot(1)	0.48	0.70

Note:

^†^Rel-Mot stands for Relationship—Motivation

In this study, prior relationship has been assigned as accounting for 10% of the simulated data (on average) and as having no difference in the use of indirect method when compared with the base level of instrumental motivation and no prior relationship (both .25). Expressive motivation has been assigned a probability of indirect method of 0.4 and on average is 26% of the data in the simulated data set. The combination of both prior relationship and expressive motivation has been assigned an average probability of indirect method of 0.7 and is allocated as 48% of the simulated sample.

### Results from simulation study

#### Using change in deviance to assess model

The simulation has been designed so that prior relationship alone should have no detectable absolute effect, expressive motivation should have a small absolute effect and the absolute effect associated with the interaction term should be the largest in magnitude and also the most detectable (due to the amount of data in this subgroup). This simulation scheme should result in the full model with main effects and an interaction term being favoured by the change in deviance test.

As Table 13 illustrates, when the sample size is large, the full model is favoured 956 times out of the 1000 simulations, with the motivation model chosen in the remaining 44 simulations (around 5%, as expected). This general pattern is repeated with a sample size of 500, but with slightly less success, in that around 77% of the simulated data sets return the full model when the change in deviance criteria is applied. However, when sample size is a modest 110, the full range of possible models is selected with reasonable frequency, with the motivation only model selected the most often. As discussed in [17], reproducibility of results is often not achievable using statistical testing (p-values) with small, noisy data. This simulation study—with all possible models being selected in multiple instances, including the null model on 23 occasions—is reflective of this phenomenon.

**Table 13:** Models chosen using change in deviance criteria.

Simulation Size	Full Model	Main Effects Model	Motivation	Relationship	Null
110	252	247	336	142	23
500	766	1	233	-	-
1000	956	-	44	-	-

**Fig 6:**
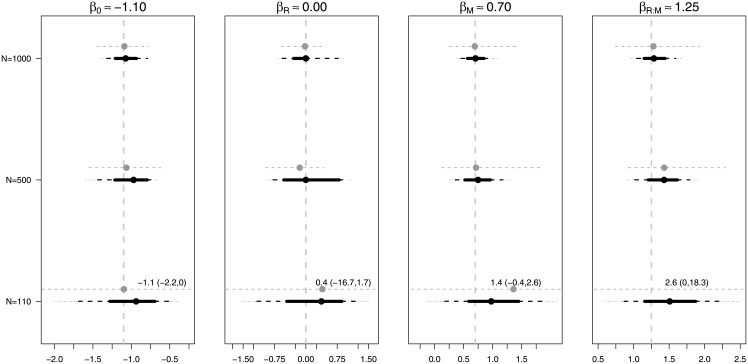
Estimated posterior mean for coefficients using BMA (black) with 20%, 50% and 95% credible intervals. Grey represents distribution of parameters from standard GLM with 95% range of estimates.

The range of estimated coefficients is illustrated in Fig 6. As expected from the results in Table 13, the coefficients for the moderate and large sample sizes are, on average, close to the true value. When the sample size is modest (110), the estimated coefficients are quite diverse, with the average of the estimates for the interaction and main effect of motivation unsatisfactory.

### Results from Bayesian Model Averaging

#### Simulated prior relationship: No effect

If we first consider the situation where we have no true effect and a small proportion of the overall data falling within this category, we note that, for the smallest sample size, which resulted in a median sample for this subgroup of 11, 50% of the simulations returned an inclusion probability of less than 0.2 (Table 14, grey column), and 75% of the simulations indicated an inclusion probability of less than 0.31. This outcome, combined with the analysts’ understanding of both the data and the research field, should lead to tenable conclusions and decisions on future research directions.

**Table 14:** Inclusion probabilities of coefficients of interest from the simulation study. Each sample size consisted of 1000 simulations. Cell sample size (*n*) differs between simulations based on the probabilities in Table 12.

Coefficient	*N*_*total*_	Median *n* (80% range*)	Inclusion probability quantiles							
			5%	10%	15%	25%	50%	75%	90%	98%
No effect (*β*_*R*_ = 0)	110	11 (7, 15)	0.08	0.09	0.11	0.14	0.20	0.31	0.48	0.79
	500	50 (41, 59)	0.00	0.00	0.04	0.05	0.10	0.22	0.48	0.89
	1000	99 (87, 113)	0.00	0.00	0.00	0.00	0.00	0.09	0.27	0.73
Moderate effect (*β*_*M*_ = .70)	110	29 (23, 35)	0.08	0.09	0.10	0.12	0.28	0.63	0.89	1.00
	500	131 (119, 144)	0.06	0.10	0.13	0.22	0.57	0.91	1.00	1.00
	1000	264 (248, 282)	0.18	0.37	0.49	0.72	1.00	1.00	1.00	1.00
Large effect (*β*_*RM*_ = 1.25)	110	52 (45, 59)	0.18	0.25	0.34	0.47	0.75	0.95	1.00	1.00
	500	237 (223, 250)	0.45	0.67	0.78	0.91	1.00	1.00	1.00	1.00
	1000	473 (455, 492)	0.79	0.92	1.00	1.00	1.00	1.00	1.00	1.00

* Represents middle 80% of simulated draws

**Fig 7:**
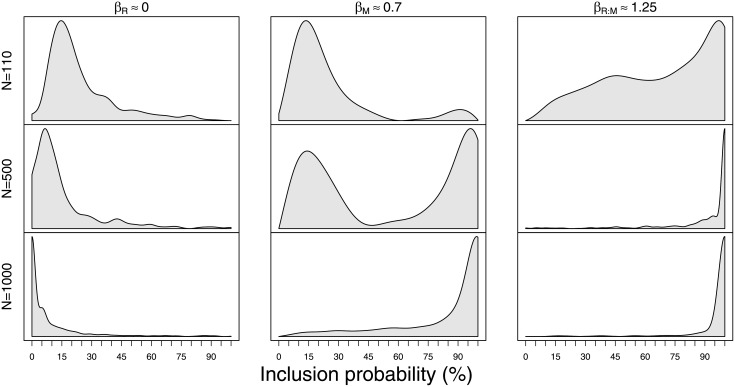
Distribution of estimated inclusion probabilities for each coefficient using data from the simulation study.

The inclusion probability of this parameter reduces dramatically as the sample size increases (Table 14; Fig 7, left column). For example, when the overall sample size is 1000, the median simulated size of this subgroup was 99. In this simulation, 50% of the BMA estimates returned an inclusion probability of zero, 75% of simulations had estimated inclusion probabilities less than 0.09, and 90% of simulations returned a value of less then 0.27. For the more moderately sized sample (500, 50 subgroup), the median inclusion probability was 0.1 with 75% of simulations having inclusion probability less than 0.22, making it unlikely that the researcher would place much credence in this effect.

Additionally, as is evidenced in Fig 6 (second column), as the sample size becomes moderate to large, the BMA coefficients closely reflect the “true” value used in the simulation. Therefore, the output of the BMA analysis tends to produce a coefficient for this absolute effect that is small in magnitude (and hence little change in relative effect) and has a small inclusion probability.

#### Simulated expressive motivation: Moderate effect

In the case of a moderate effect size, Table 14 indicates that, with a small sample size, 50% of the simulations returned an inclusion probability of 0.28 or lower. The inclusion probability improves considerably to 0.57 for 50% of the simulations with a moderate sample size and than to 1.0 when the sample was large. With a large sample size, 85% of the simulations resulted in an inclusion probability greater than 0.5.

Fig 7 (middle column), shows this transition with very few simulations returning large inclusion probabilities when the sample size is small. The situations results in a bi-modal distribution at moderate sample size, with around 25% of the simulations indicating an inclusion probability of 0.9 or greater (see Table 14 and Fig 7). With a large sample size, 85% of the simulations resulted in an inclusion probability greater than 0.5. In terms of accuracy of the estimated coefficients, once again, at moderate and large sample sizes, the mean estimated coefficients from the simulation are close to the simulated value (see Fig 6), with a small 95% credible range. The results of the simulation study for moderate absolute effect size show that researchers who incorporate their sample size knowledge along with the inclusion probability from BMA are likely to make sensible inferences and future planning for research.

#### Simulated expressive motivation and prior relationship: Large effect size

When the effect size is large, as Table 14 indicates, 50% of simulations at all three sample sizes returned high inclusion probabilities. For the small sample size, 50% of the simulations resulted in an inclusion probability of at least 0.75. At the large sample size, all simulations returned high inclusion probabilities, with 95% of the estimates greater than 0.79. This simulation indicates that researchers are likely to make tenable inferences when using BMA for logistic regression when absolute effects are large, regardless of sample size.

#### Comparison of change in deviance and BMA

**Fig 8:**
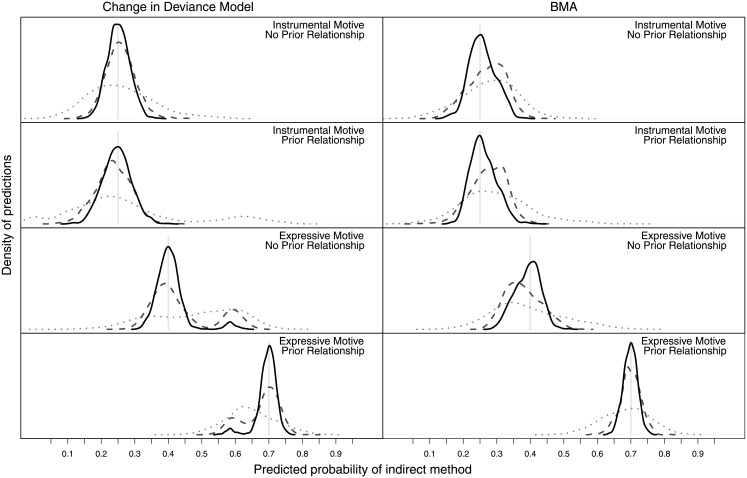
Density of predictions based on models using change in deviance (left) and BMA (right). Solid black line represents results *N* = 1000, medium grey dashed line *N* = 500, and light grey dotted line is *N* = 110. True population proportion indicated by grey vertical line.

Fig 8 is a summary of the predictions for each scenario from the simulation study. At the small sample size, the change in deviance estimates is clearly inaccurate, particularly for the main effect variables. In terms of reproducibility, this technique would not be advisable for small samples. For medium and large samples, estimates from the change in deviance improve, however, there are cases where the moderate effect (expressive motivation) is substantially overestimated and the largest effect (interaction) is underestimated. From Fig 8, we note that predictions from BMA are relatively robust to sample size, improving as sample size increases. There is no evidence of aberrant behaviour for the moderate and large effects in BMA as seen in a small number of cases (around 5%) with the change in deviance model choice.

Overall, BMA indicates that the coefficient with zero effect is not required in the model (inclusion probability near zero) for moderate and large samples, and has low inclusion probabilities at the small sample size, allowing researchers to consider the effect in future studies. Moderate and large effects result in larger inclusion probabilities for all sample sizes, and these become more stable with increasing sample size. In contrast, the change in deviance criteria performs poorly with a small sample size, and results in the full model at the larger sample size due to the need for the interaction term designed within the simulation study, however, in around 5% of the analyses conducted using this model fit approach, we would expect unsatisfactory inferences.

## Discussion and conclusion

The purpose of this paper was to encourage our readers to move away from focusing on testing the effects toward the idea of understanding the statistical model and the inferences that can be drawn from an analysis. Advances in statistical software—in particular, the free software R [46]—are enabling researchers to use modern statistical modelling approaches for their analyses, which tend to provide far greater insight and understanding of the data obtained in research than statistical testing alone. This capacity is further enhanced by the ease with which researchers can visualize their data and perform model diagnostics.

The objective of this paper was first to show that GLMs with explicitly specified interactions produce results that are difficult to interpret using conventional strategies based on significance testing using the Wald statistic and change in deviance criteria. We then proposed and examined strategies of Bayesian estimation using vague priors and Bayesian Model Averaging. Table 15 presents a brief comparison of the benefits and tenable inferences that can be drawn using each of the four approaches examined in this paper.

**Table 15:** Comparison of statistical techniques and their main benefits.

Benefits	Wald test	Change in deviance	Bayesian GLM	BMA
Hypotheses: Main effects	Y	Y	Y	Y
Hypotheses: Interactions	N	N	Y	Y
Suitable for small samples	N	N	Y	Y
Not affected by coding contracts	N	N	Y	Y
Accounts for model uncertainty	N	N	N	Y
Software available	Y	Y	Y	Y

The Wald test is well known, often used in the literature, and easy to access, as it is included in the model summary output in most statistical packages. However, it has a number of limitations, one of which is that it relies on large samples, which are not always possible in social sciences, especially in new areas of research or when difficult to access populations are involved. Furthermore, it performs best when the effect sizes are small to medium, as it can demonstrate aberrant behaviour when the effects are large. Additionally, results produced by the Wald test are affected by coding contrasts, and researchers need to be mindful of this, as different coding contrasts could lead to a different interpretation of the effects. Because of these limitations, when the Wald statistic is being used, the absence of significance does not necessarily indicate that there is no effect—in particular, in small or medium samples or when we are dealing with very strong effects.

Another method of measuring the effects, change in deviance, is also readily understood, often cited in the literature, and accessible in most statistical packages. However, similar to the Wald statistic, it depends on large samples. As the purpose of these statistics is to find the balance between model fit and parsimony, it tends to be conservative, and often underestimates the need for interactions in the model.

Unlike the Wald test and the change in deviance method, Bayesian GLM with vaguely informative priors is not based on statistical testing using large sample asymptotics. Bayesian GLM is highly applicable when the researcher has some idea of the direction of effects—for example from previous research—or, at the very least, has a good understanding of the model and plausible effect sizes. But in new ares of research or for analysts new to Bayesian concepts, selecting appropriate priors may be difficult. In this situation, analysis may be conducted using uninformative priors. The point estimates and resulting summary from such a model will mirror standard techniques; however, interpretations will be valid with small samples and the researcher will have the added advantage of the rich information contained in the posterior distributions.

Bayesian Model Averaging allows a fuller understanding of model fit and parameter/effect size, direction and importance, in situations where we are looking for an indication of potential effects rather than absolute certainty of significance (i.e. prediction is not the main goal). Furthermore, BMA accounts not just for parameter uncertainty, but also for model uncertainty, which makes it especially suitable for new areas of research when the theory is weak or not yet available [21]. BMA offers guidance in terms of variables selection and model building when information is scant. BMA allows researchers to make informed decisions about whether to use one “best” model or to average predictions over a number of models to estimate posterior distributions for model coefficients.

Our example study demonstrated that some techniques, such as converting binary variables into a single multi-level factor or computing posterior estimates using a Bayesian framework, can be useful in small-dimensional models. However, for larger-dimensional models, we recommend BMA analysis should be a routine tool used in any modelling strategy. It is robust to the dimension of the model and is relatively straightforward to implement and interpret. The code supplied in this paper illustrates that using the BMA library in R [51] is no more difficult than implementing the basic glm(). We suggest that the BMA package is suitable to perform initial data analysis as it produces a set of best models and evaluates the contribution of each individual coefficient.

As variable selection and model averaging are highly active areas in statistical research, there are multiple options from which researchers can choose in conducting such an analysis, many of which are implemented in R libraries. For example, the BAS package [53] provides the flexibility of a fully Bayesian model using a number of options for model priors, predictions, credible intervals and various diagnostic and inferential plots. However, it requires some additional expertise in the use of priors, so may be better used at more advanced stages of the analysis, or as analysts become more comfortable with the techniques. The required level of expertise is not beyond the reach of researchers, and is well documented in [54] and [55].

The growth in options for researchers wishing to conduct statistical analysis using BMA is rapid. When Amini and colleagues (2011) [44] conducted a review of BMA libraries in R there were three (3) official libraries available on CRAN: BAS [53], BMA [51] and BMS [56]. Current libraries available through CRAN, which can be viewed at https://cran.r-project.org/view=Bayesian, include updated versions of these packages, and in addition, libraries such as mlogitBMA [57], BayesVarSel [58] and BoomSpikeSlab [59]. The use of these packages removes impediments to the routine implementation of BMA as a standard tool for social scientists.

## Supporting information

S1 File Interview schedule. (DOCX)

S2 File Dataset. (CSV)

S3 File R code. (R)
