# Supplementary material for: Non-significant p-values? Strategies to understand and better determine the importance of effects and interactions in logistic regression
Source: PLoS One. 2018 Nov 26;13(11):e0205076. doi: 10.1371/journal.pone.0205076 (PMC6261058; doi:10.1371/journal.pone.0205076)
Supplement: S1 File — (DOCX) [file pone.0205076.s001.docx]

**Victim Interview Schedule**

Q1: Please tell us about yourself (age in years, gender, highest educational attainment, occupation and employment status.

Q2: Please describe yourself as an Internet user – do you consider yourself an active Internet user; how often do you access the Internet; do you own a mobile phone; do you access the Internet via your mobile phone, if yes, how often; do you use social media, if yes, how often; how many social media accounts do you have; do you play online games, any other online activity related details you wish to share?

Q3: You mentioned that you have experienced at least one incident of cyber abuse in some form in the past. Thinking of the most recent or most memorable incident, please describe what happened. How did the incident begin?

Q4: What were you doing when the attack happened?

Q5: What do you think caused the incident?

Q6: Did you know the person(s) who attacked you? What was the nature of your relation-ship with the offender?

Q7: What happened afterwards?

Q8: How long did the incident continue for? Did you resolve the incident? How?

Q9: How did you react to this incident? What effect (if any) did this incident have on your life?
